# Food Service Directors’ Knowledge and Beliefs About Ultraprocessed Foods in California’s San Joaquin Valley Schools

**DOI:** 10.5888/pcd23.250379

**Published:** 2026-07-09

**Authors:** Anjali Gupta, Alix Zuceth Duran Gomez, Donna Matheson, Aditi Sharma, Viviane Richard, Lorrene D. Ritchie, Ashley de la Rosa, Deiglis Delgado, Genoveva Islas, Christina A. Hecht, Kenneth Hecht, Anisha I. Patel, Andrea Pedroza-Tobias

**Affiliations:** 1 Stanford University School of Medicine, Stanford, California; 2 Department of Pediatrics, Stanford University School of Medicine, Palo Alto, California; 3 Division of Primary Care, Geneva University Hospitals, Geneva, Switzerland; 4 Nutrition Policy Institute, University of California, Agriculture and Natural Resources, Oakland, California; 5 Dolores Huerta Foundation, Bakersfield, California; 6 Cultiva La Salud, Fresno, California

## Abstract

**Introduction:**

Ultraprocessed foods (UPFs) are industrial formulations of ingredients typically not used in home cooking and often high in added sugars, saturated fat, or sodium. UPF intake is associated with obesity, type 2 diabetes, hypertension, and other chronic diseases. Although the National School Lunch Program and School Breakfast Program must meet US Department of Agriculture nutrition standards, federal standards do not specifically address food processing. However, reducing UPFs in schools is gaining momentum. As food service directors (FSDs) are key implementers of school nutrition policies, we sought to characterize their knowledge and beliefs about UPFs in school meals.

**Methods:**

In this qualitative study, we conducted 20 semistructured interviews with FSDs in California’s low-income, largely rural San Joaquin Valley from January through June 2024. Two analysts double-coded transcripts and used inductive thematic analysis to identify key themes.

**Results:**

Of 20 FSDs, 16 completed the demographic survey. Participants’ mean age was 49.5 years (IQR, 41.0–59.5), 15 were women, 8 identified as White, and 6 identified as Hispanic or Latino. Seven FSDs reported that they had never heard of UPFs. After receiving a definition, FSDs generally described UPFs as less healthy than scratch-cooked foods, although several perceived UPFs served in schools as healthier than UPFs sold elsewhere. FSDs cited student food insecurity, food safety concerns, and student acceptance as reasons for serving UPFs; others worried that serving UPFs at school could reinforce unhealthy eating habits.

**Conclusion:**

Training that is free of conflicts of interest and food labeling systems that are clearer may improve FSDs’ ability to identify UPFs and implement policies to reduce UPFs in school meals.

**Summary**

**What is already known on this topic?**

Consumption of ultraprocessed foods is associated with chronic diseases. Food service directors are critical in implementing emerging school policies related to ultraprocessed foods.

**What is added by this report?**

Seven of 20 food service directors in California’s San Joaquin Valley said they had never heard the term ultraprocessed foods. Some food service directors also expressed misconceptions about ultraprocessed foods, including their healthfulness.

**What are the implications for public health practice?**

Training that is free of conflicts of interest and food labeling systems that are clearer may improve food service directors’ ability to identify and implement policies to reduce ultraprocessed foods in school meals.

## Introduction

Ultraprocessed foods (UPFs) are industrial formulations of ingredients typically not used in home cooking and often high in added sugars, saturated fat, or sodium (1). In 2018, UPFs accounted for 67.0% of total energy intake among US youth aged 2 to 19 years (2).

Systematic reviews and meta-analyses show that UPF intake is associated with lower diet quality and chronic disease outcomes, including cardiovascular disease, type 2 diabetes, hypertension, dyslipidemia, and obesity (3–5). Health associations may differ across UPF subgroups, highlighting the need for rigorous studies free of conflict of interest (6). Proposed mechanisms extend beyond nutrient profile and include exposure to packaging-related chemicals, disruption of the food matrix, and effects of cosmetic additives on inflammatory pathways and gut microbiota (7).

Because children and adolescents spend much of their day at school, school nutrition programs offer a practical setting for reducing UPF exposure. The National School Lunch Program and the School Breakfast Program (hereinafter, “school meals”), administered by the US Department of Agriculture, provide free or low-cost meals to eligible students from low-income backgrounds (8). In 2010, the Healthy, Hunger-Free Kids Act (9) increased school meal nutrition requirements for fruits, vegetables, and whole grains; limited total calories, sodium, and total fats; eliminated trans fats; and established standards for all competitive foods sold in schools. However, current federal school nutrition standards do not classify or limit foods by degree of processing; consequently, products that meet nutrient standards can still be UPFs (10).

Policy interest in limiting UPFs in school meals is increasing. At least 10 states have introduced legislation to reduce UPFs in school meals (11). In October 2025, California enacted the Real Food, Healthy Kids Act (Assembly Bill 1264), which requires the California Department of Public Health to define “restricted school foods” and “ultraprocessed foods of concern” by June 1, 2028; schools must begin phasing out those foods by July 1, 2029; vendors may not offer them to schools beginning July 1, 2032; and nutritionally adequate school breakfasts and lunches may not include them beginning July 1, 2035 (12).

Food service directors (FSDs) are critical in implementing school nutrition policies and guiding what is served in schools. However, to our knowledge, no studies have examined their understanding of UPFs. We sought to characterize FSDs’ knowledge and beliefs about UPFs in school meals.

## Methods

### Study design

Data for this analysis came from a broader semistructured interview study with FSDs in California’s San Joaquin Valley that explored perceived barriers and facilitators to providing freshly prepared meals and reducing UPFs in school meals.

The study was designed by a community–academic partnership among 2 San Joaquin Valley community-based organizations (Dolores Huerta Foundation and Cultiva La Salud) and 2 academic institutions (Stanford University and the University of California Nutrition Policy Institute). We convened a community advisory board to provide input on all study processes.

### Study population

This study was conducted in California’s San Joaquin Valley, a predominantly Hispanic/Latino, rural, and low-income region that produces 8% of the nation’s agricultural food (13). To recruit participants, we first obtained demographic information about the 185 San Joaquin Valley school districts from publicly available California Department of Education records (14,15). We also recorded whether districts received state Kitchen Infrastructure and Training Freshly Prepared Meals grant funding, available to districts that attested that at least 40% of their school meals were freshly prepared (15).

The school districts were stratified into 4 groups based on receipt of the Freshly Prepared Meals grant (yes/no) and geographic locale (rural/urban) (16) to ensure representation across strata. Within each stratum, districts were randomly ranked. We initially emailed the first 10 FSDs per stratum and followed up with nonrespondents by telephone. Recruitment proceeded iteratively; as interviews were conducted, we reviewed emerging themes and continued contacting additional FSDs from the randomized lists until thematic saturation was achieved. In total, 43 FSDs were contacted and 20 consented to participate (46%) (Figure).

**Figure:**
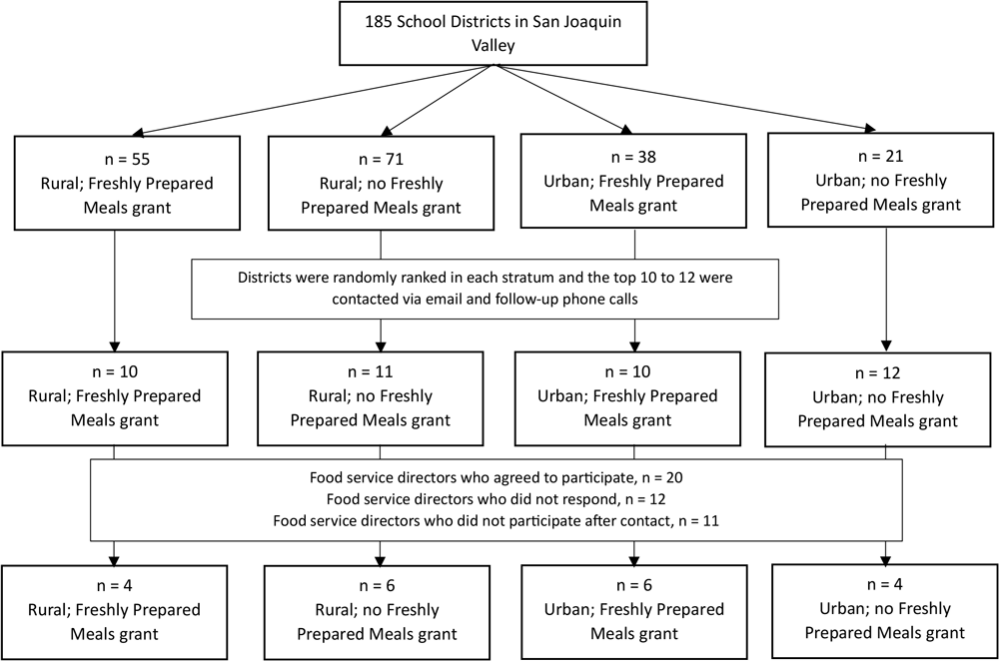
Recruitment process for food service directors, San Joaquin Valley, California, 2024. Following National Center for Education Statistics classification, rural and town locales were classified as “rural” and suburban and urban locales as “urban.” Supplementary state-level Kitchen Infrastructure and Training funding was granted to districts that attested that at least 40% of their school meals are freshly prepared onsite (Freshly Prepared Meals grant).

### Interview guide

The study team designed the semistructured interview guide (https://researchinchildhealth.org/resources-ultra-processed-foods-in-school-meals) with input from the community advisory board. The guide covered topics such as the FSD role; school meal planning and purchasing processes; perceptions of school meal healthfulness; knowledge and beliefs about UPFs; barriers and facilitators to increasing scratch-cooked meals (ie, meals with minimally processed foods); reducing UPFs; and students’ acceptance of scratch-cooked meals. The current analysis focused on interview responses regarding FSDs’ knowledge and beliefs about UPFs. For the analysis of FSDs’ initial understanding of UPFs, participants were asked, “Have you ever heard of ultraprocessed foods?” and/or “What comes to mind when you think of ultraprocessed foods?” Before discussing beliefs about UPFs, researchers presented a slide that defined UPFs and provided examples to ensure a common understanding. The guide was pilot-tested with 2 FSDs who were members of the community advisory board.

### Data collection

Semistructured interviews (45–60 minutes each) were conducted with 20 FSDs from January to June 2024. Participants reported sociodemographic data (age, sex, race, and ethnicity) in a 10-minute survey. Two female researchers conducted the interviews via Zoom. Dr Pedroza-Tobias is a bilingual (Spanish/English) Latina postdoctoral scholar (MSc, Nutrition; PhD, Global Health) and Dr Matheson is a White senior research scientist (MSc and PhD, Nutrition). Both interviewers were mothers of children in public schools. The researchers had no prior interactions with participating FSDs. Verbal consent was obtained from participants. Participants who spoke either English or Spanish were invited to participate; all chose to be interviewed in English. FSDs could invite a team member to participate with them if they could provide insight on the topic. All interviews were conducted with a single study participant except for 2 interviews: one included a cafeteria aid, and another included a child nutrition coordinator. No interviews were repeated. Interviews were video- and audio-recorded, transcribed verbatim, and deidentified. Transcripts were not returned to participants for correction. However, a written summary of preliminary findings was emailed to all participating FSDs for their feedback on the accuracy of the interpretations; no participants responded. Findings were also shared with the community advisory board, and the study team incorporated feedback. Participants were offered $50 gift cards for their participation. Three FSDs declined the incentive. The Stanford Institutional Review Board approved the study (IRB no. 70983).

### Analysis

Participant demographic characteristics were summarized with medians and IQRs for continuous variables and frequencies and percentages for categorical variables. Qualitative data were analyzed inductively by using thematic analysis. The study team discussed emergent themes after each interview. Data saturation was reached after 20 interviews, when no new themes were observed. Two analysts (A.P.-T. and A.G., a female medical student who attended US public schools) reviewed the interviews to identify emergent topics and reached consensus through discussion. An initial codebook was developed. The analysts then double-coded all interviews, resolving discrepancies through discussion, adding new codes, and refining the codebook as necessary. Reflexivity was maintained by discussing potential biases and perspectives among the research team. Codes were organized into a coding tree (Appendix) to illustrate the relationships among codes and themes. NVivo 12 (Lumivero) was used to organize codes. Quotes have been lightly edited to remove vocal disfluencies (eg, fillers such as “um” or “like”).

## Results

### Participant demographics

Of 20 participants, 16 completed the demographic survey. Geographic locale and Freshly Prepared Meals grant status were known for all participants; among the 16 who completed the demographic survey, 7 were from rural districts and 9 were grant recipients. Among survey respondents, the median age was 49.5 years (IQR, 41.0–59.5); 15 identified as women, 8 as White, and 6 as Hispanic (Table). Six of 16 survey respondents estimated that more than 40% of foods purchased for school meals were UPFs.

**Table:** Characteristics of Food Service Directors Who Completed the Demographic Survey (N = 16), San Joaquin Valley, California, 2024^a^

Characteristic	Value
Age, median (IQR), y	49.5 (41.0–59.5)
Female sex	15 (94)
**Race and ethnicity**	
Hispanic or Latino	6 (37)
White	8 (50)
American Indian	1 (6)
≥2 Races	1 (6)
**Other**	
Rural^b^	7 (44)
Recipient of Freshly Prepared Meals grant^c^	9 (56)
Days per school week doing any scratch cooking,^d^ median (IQR)	2.0 (1.5–3.0)
Estimated that >40% of foods purchased for school meals were UPFs^e^	6 (37)

Abbreviation: UPFs, ultraprocessed foods.

a Of the total sample of 20 participants, 4 did not complete the demographic survey. Values are n (%) unless otherwise indicated.

b Following National Center for Education Statistics classification, rural and town locales were classified as rural, and suburban and urban locales were classified as urban.

c Supplementary state Kitchen Infrastructure and Training funding granted to districts that attested that at least 40% of their school meals are freshly prepared onsite (Freshly Prepared Meals grant).

d Response obtained from the following question: “In a typical week, how many days at a typical school does your School District use each of the following for reimbursable meals? School-made/scratch or modified scratch preparation (use of minimally processed foods, some degree of ingredient preparation and cooking when needed, eg, spaghetti with scratch-prepared sauce).

e UPFs were defined for participants as industrial formulations of ingredients that typically contain excess sugars, oils and fats, and salt.

### Knowledge and understanding of ultraprocessed foods

Before presenting a common definition of UPFs, participants were asked, “Have you ever heard of ultraprocessed foods?” and/or “What comes to mind when you think of ultraprocessed foods?” In response, 7 of 20 FSDs reported being unfamiliar with UPFs: “Ultra processed, maybe processed a little bit more to pull in more nutrients, maybe? I don’t know. I would think that they wouldn’t want to process it and take more nutrients out” (ID 20). Of the 7 FSDs who expressed unfamiliarity with UPFs, 6 were Freshly Prepared Meals grant recipients, and 3 were from rural districts. The remaining 13 FSDs described UPFs as packaged foods containing additives and preservatives. For example, one FSD said: “I would define it as . . . they’ve taken . . . wheat and bleached it and added chemicals to preserve it. And, then the same thing with a meat product, they’ve taken it, processed it, added some kind of preservative, flavoring, color to it, and . . . prepackaged it into a food-like product” (ID 14). Another said: “It’s like food that has a lot of chemicals and a lot of stabilizers. And typically they’re often shelf stable” (ID 10). Some described UPFs as foods altered from their natural form: “Breaded nuggets, and you’d eat them, and they were like rubber, and I was like, what is it? There isn’t even any chicken in these things” (ID 07).

### Perceptions of ultraprocessed foods

The following themes reflect FSDs’ perceptions of UPFs after researchers presented a common definition and examples.

#### Perceived healthfulness of ultraprocessed food

When asked about the healthfulness of UPFs, more than half of FSDs characterized UPFs as less healthy than scratch-cooked foods, although their understanding of potential health effects varied. Some FSDs said they thought UPFs could adversely impact health. For example, one FSD said, “It’s not good for your body, it causes . . . cancer, illnesses, obesity. And honestly, I think some of that ultraprocessed food, when you eat it, makes you more hungry. At least, for me and other people I’ve seen, ’cause you’re not nourishing your body. . . . It’s going to crave more food” (ID 07). Some also thought UPFs could affect learning: “I tend to believe that it is a contributing factor to food addiction, obesity, and the ever-growing ADHD epidemic of kids not being able to pay attention, focus, and generally not learn” (ID 19).

Two FSDs said UPFs were not necessarily less healthy than scratch-cooked foods. One said, “I don’t think it’s unhealthy. I think as long as they’re not getting 5 slices of pizza, it’s just everything in moderation” (ID 11). Several FSDs perceived that UPFs served in schools were healthier than UPFs served outside schools: “And you can get a Wild Mike [pizza] at the grocery store, but the one we’re serving . . . it’s more nutritious because it’s governed by the state to be nutritious for school food services” (ID 20). In contrast, one FSD explained that they did not believe UPFs in schools were healthier than those elsewhere, but regulations legitimize the serving of certain UPF products in school: “You know, I get to simply say, ‘Oh, no, it’s Smart Snack compliant, you know the State says so.’ I’m grateful, I guess that I have that sort of safeguard there, that it isn’t me. I’m just here to uphold these regulations, but I personally think that it’s marginally different” (ID 14). In addition, one FSD believed that foods prepared more recently were fresh and less processed: “[Domino’s pizza is] also a fresher product, you know, they’re building it morning of and cooking it. So, it’s not something that’s been sitting in the freezer for months” (ID 01).

#### Perceived taste of ultraprocessed foods compared with scratch-cooked foods

Most FSDs said scratch-cooked foods tasted better than UPFs. For example, one FSD noted that scratch cooking provides flexibility to adapt recipes to students’ tastes: “I think scratch cooking is better just because one, it gives you freedom to kind of build what you want your recipe to be or even build it to your students. So, I know that all of my schools, all the students, like different things. So, when my cooks are building their recipes . . . this school over here really likes a spicy spaghetti and so they manipulate the recipes to go and gear towards their students” (ID 05).

#### Reasons for serving ultraprocessed foods

FSDs described several reasons for serving UPFs. One felt that food safety takes priority: “It’s eggs or chicken. . . . Those would be the things that I would just worry about. Anything that creates more of a foodborne illness or an allergy . . . those are the things that are kind of scary to put in your kitchens” (ID 05). Several FSDs said ensuring that students ate at school took priority because of high food insecurity in the San Joaquin Valley; many noted that school meals were a major source of sustenance for their students. For example, one FSD said, “I do serve some ultraprocessed food. . . . And certainly, I feel that they need to eat, and if they’re eating it, then you know that they’re doing good. . . . And a lot of kids, you know, this is the only place they do eat. So, I want to make sure that I’m giving them something that they will eat” (ID 09).

#### Commitment to reducing UPFs in school meals

Many FSDs described their role as providing students with high-quality nutrition. For example, one FSD explained that cooking healthy food requires more time and effort but brings great reward: “And you can walk away through the day feeling good that you know those kids ate well. And we’ve done our best to fuel them, not just feed them” (ID 19). Multiple FSDs said they read food labels when deciding which foods to purchase: “What’s important is that people who are cooking this food that they do read the . . . they print out the nutritionals [nutritional data] and they look at them” (ID 13).

#### External influences shaping beliefs about ultraprocessed foods

Many FSDs mentioned external influences that shaped their beliefs about UPFs, including public discussion of UPFs, family roles and household experiences, and marketing. With respect to public awareness, one stated: “Science is backing up with evidence, and that evidence is getting disseminated to the general population. We’re finally catching up that we’ve been basically ‘quote unquote’ poisoning ourselves all these years with some of these chemicals that we’ve been eating” (ID 10). Furthermore, several FSDs brought up their families when thinking about UPFs: “I think especially being a mother, you know, I take my job a little bit more serious now” (ID 08). Lastly, several FSDs mentioned marketing from UPF brands and the ready accessibility of UPFs: “And it’s because a lot of times, that people make it so easy . . . grocery stores or outside of our schools, like they [food vendors] don’t park right outside [the school] but they’re right at the park, where they have those hot Cheetos and all that” (ID 08).

## Discussion

School meal programs, which serve nearly 30 million children each school day (8), provide an important setting for reducing exposure to foods associated with chronic disease risk (3–5) associated with UPF consumption. In semistructured interviews with 20 FSDs in a predominantly agricultural, low-income, rural region of California, we found varying knowledge and beliefs about UPFs.

A notable finding was that 7 of 20 FSDs had not heard the term “ultraprocessed foods.” This unfamiliarity occurred even among FSDs in districts that had dedicated Freshly Prepared Meals grant funding to support fresher, less-processed school meals. This highlights a gap in FSDs’ awareness and understanding of the UPF concept. Although FSDs must follow federal school nutrition guidelines (17), UPFs that have been reformulated to meet these guidelines are commonly served in schools. Therefore, providing training on UPFs that is free of conflicts of interest (18) could increase FSDs’ knowledge about UPFs. Training could include a UPF definition, guidance on reading ingredient and nutrition labels, and practical strategies for identifying UPFs and choosing less-processed options. To increase feasibility, training should be brief and incorporated within existing paid working hours or district-supported staff development time. This training can also be coupled with funding targeted toward increasing scratch cooking in school meals.

Our finding that one-third of FSDs were unfamiliar with UPFs highlights ongoing confusion and debate about the UPF definition in academic and public contexts (19). Thus, using a consistent UPF definition, such as the NOVA classification (20) or the definition developed under California’s Real Food, Healthy Kids Act (12), along with concrete examples and plain-language guidance, may be an important step in raising awareness.

In addition, front-of-package labeling systems have been implemented in various countries to identify products high in nutrients of concern or containing ingredients such as nonnutritive sweeteners. The US Food and Drug Administration has proposed a front-of-package Nutrition Info box that would identify whether saturated fat, sodium, and added sugars are present in low, medium, or high amounts. Future research and policy development could examine whether adding UPF information to front-of-package labeling systems helps FSDs identify UPFs (21).

After participants received the study’s UPF definition, many of them perceived UPFs available in schools as healthier than UPFs available at fast-food restaurants and grocery stores. As noted, the Healthy, Hunger-Free Kids Act prompted reformulation of school food products to meet the US Department of Agriculture’s nutrition standards, including the incorporation of whole grain–rich and lower sodium products (22). Although there is heterogeneity in health associations among UPF subgroups (23), and some food products reformulated for schools may contain lower sodium, fat, and calories, or more whole grains than similar items sold elsewhere, these “healthier” reformulated items may legitimize the consumption of UPFs (24). As one FSD described, certain UPFs are “Smart Snacks compliant,” making it easier to justify including these foods in schools. These foods also enable manufacturers to build brand loyalty among millions of children (25) and may encourage consumption of less healthy versions of reformulated products available outside of school (22). Given these concerns, there is substantial policy interest (eg, more stringent federal nutrition standards, school district procurement policies) in limiting access and availability of UPFs in schools (26–28).

FSDs described several reasons why they serve UPFs to students. One FSD explained that cooking with raw ingredients, such as chicken, raises concerns about foodborne illnesses from improper handling or cooking. Processed heat-and-serve meat products are typically precooked, which may reduce perceived food safety risks but does not eliminate the need for safe handling (29). Therefore, to reduce UPFs in schools, FSDs and kitchen staff may need food safety training and enhanced kitchen infrastructure to safely handle raw ingredients. Another reason FSDs gave for serving UPFs was concern that children, particularly those experiencing food insecurity, might not eat school meals and would face hunger. This may be a particular concern in communities like the San Joaquin Valley, where a large proportion of families face food insecurity and may be more reliant on school meals (30).

Prior research is consistent with our finding that FSDs view student taste preferences as a barrier to increasing the healthfulness of school meals (31,32). In a study of more than 250 US FSDs, 75% indicated that “pressure to serve foods that children enjoy versus healthy foods” was a barrier to improving healthy food choices in the school food environment (31). This may be due to students having less exposure to unprocessed foods at home or other out-of-school settings because of cost and convenience barriers to less-processed options. However, some evidence suggests that students may prefer fresh, less-processed foods when these foods are acceptable, appealing, and feasible within school meal programs (33,34). Given these discrepancies, reducing UPF access in schools may require education and discussion among all members of the school community (eg, students, parents, teachers, staff).

### Limitations and strengths

This study has several limitations. First, our sample was restricted to FSDs in California’s San Joaquin Valley. California is unusual in its universal school meals program, and the San Joaquin Valley is a largely low-income, rural agricultural region (13). As with most qualitative studies, findings are not intended to be statistically generalizable. Rather, our goal was to provide sufficient contextual detail to allow readers to assess transferability of findings to other settings and inform larger studies. Second, the potential for social desirability bias exists. Third, we provided a definition and examples of UPFs before asking about beliefs to ensure a common understanding, because several FSDs were unfamiliar with the term at the start of the interviews. Although it is possible that later statements about FSDs’ perceptions of UPFs were influenced by what was presented, we elicited participants’ initial knowledge of UPFs before presenting this definition. Fourth, conducting interviews via Zoom may have influenced participation and interaction patterns. Fifth, while further comparative analysis across contexts, such as geography and income level, could be beneficial in advising policy, this was not possible given the small sample size. Strengths include the range of FSD perspectives obtained through stratified recruitment and the study’s contribution to an emerging literature on FSD knowledge and beliefs about UPFs.

### Conclusions

FSDs in this study had varying understandings of UPFs: 7 of 20 had not previously heard the term, whereas others described UPFs in ways consistent with common definitions, including packaged foods containing additives or preservatives and foods altered from their natural form. Given growing interest in reducing UPFs in schools, training, education, and improved front-of-package labeling systems may help FSDs understand and recognize UPFs. It will also be important to engage families to ensure that introducing less-processed items does not decrease participation in school meals, particularly in communities facing food insecurity. These strategies may help improve the quality and healthfulness of school food and support the implementation of policies aimed at reducing UPFs.

## Appendix Qualitative Analysis Coding Tree

**Table:**

Theme	Example codes
Understanding of the term ultraprocessed food	• Never heard of the term • Accurate understanding (eg, packaged, preservatives, additives, unrecognizable ingredients)
Healthfulness of ultraprocessed food	**•** Ultraprocessed food is less healthy than unprocessed foods • Ultraprocessed food is equally healthy or healthier compared with unprocessed foods • Ultraprocessed food served in schools is healthier than that served outside of school
Taste of ultraprocessed food	**•** Ultraprocessed food is less tasty than unprocessed foods • Ultraprocessed food is equally tasty or tastier compared with unprocessed foods
Reasons for serving ultraprocessed food in school meals	• Concerns related to food safety and foodborne illness • Concerns related to student consumption given high food insecurity among students
Commitment to reducing UPFs in schools	• Work satisfaction from serving healthy foods • Importance of helping students build healthy habits
External influences shaping beliefs regarding ultraprocessed food	• Food culture in America is becoming more preservative-free, with less processing • Influences from their own families • Marketing from ultraprocessed food brands
